# Genome scan study of prostate cancer in Arabs: identification of three genomic regions with multiple prostate cancer susceptibility loci in Tunisians

**DOI:** 10.1186/1479-5876-11-121

**Published:** 2013-05-13

**Authors:** Jingxuan Shan, Khalid Al-Rumaihi, Danny Rabah, Issam Al-Bozom, Dhanya Kizhakayil, Karim Farhat, Sami Al-Said, Hala Kfoury, Shoba P Dsouza, Jillian Rowe, Hanif G Khalak, Shahzad Jafri, Idil I Aigha, Lotfi Chouchane

**Affiliations:** 1 Laboratory of Genetic Medicine and Immunology, Weill Cornell Medical College in Qatar, Qatar Foundation, Doha, Qatar; 2 Department of Surgery, Hamad Medical Corporation, Doha, Qatar; 3 Princess Al-Jouhara Al-Ibrahim Cancer Research Center, College of Medicine and King, Khalid University Hospital, King Saud University, Riyadh, Saudi Arabia; 4 Department of Laboratory Medicine and Pathology, Hamad Medical Corporation, Doha, Qatar; 5 Information Technology Services Division, Weill Cornell Medical College in Qatar, Qatar Foundation, Doha, Qatar

**Keywords:** Prostate cancer, GWAS, Arab population

## Abstract

**Background:**

Large databases focused on genetic susceptibility to prostate cancer have been accumulated from population studies of different ancestries, including Europeans and African-Americans. Arab populations, however, have been only rarely studied.

**Methods:**

Using Affymetrix Genome-Wide Human SNP Array 6, we conducted a genome-wide association study (GWAS) in which 534,781 single nucleotide polymorphisms (SNPs) were genotyped in 221 Tunisians (90 prostate cancer patients and 131 age-matched healthy controls). TaqMan^®^ SNP Genotyping Assays on 11 prostate cancer associated SNPs were performed in a distinct cohort of 337 individuals from Arab ancestry living in Qatar and Saudi Arabia (155 prostate cancer patients and 182 age-matched controls). *In-silico* expression quantitative trait locus (eQTL) analysis along with mRNA quantification of nearby genes was performed to identify loci potentially *cis*-regulated by the identified SNPs.

**Results:**

Three chromosomal regions, encompassing 14 SNPs, are significantly associated with prostate cancer risk in the Tunisian population (*P* = 1 × 10^-4^ to *P* = 1 × 10^-5^). In addition to SNPs located on chromosome 17q21, previously found associated with prostate cancer in Western populations, two novel chromosomal regions are revealed on chromosome 9p24 and 22q13. eQTL analysis and mRNA quantification indicate that the prostate cancer associated SNPs of chromosome 17 could enhance the expression of *STAT5B* gene.

**Conclusion:**

Our findings, identifying novel GWAS prostate cancer susceptibility loci, indicate that prostate cancer genetic risk factors could be ethnic specific.

## Introduction

Prostate cancer (PCa) is the most common malignancy in western countries and the second cause of cancer-related death in Europe and the United States [1]. With lifestyle changes, the incidence of the disease has been increasing in the Arab populations [2]. From 1991–2006, prostate cancer was ranked first among cancers in Qatari males over 65 years old [3]. In Kuwait, the incidence of prostate cancer rose to 12.3/100,000 men/year in 2004 [4]. In 2003, prostate cancer was ranked as the fourth most diagnosed cancer in Tunisia [5]. In Lebanon, the age-adjusted standardized incidence was 21.5/100,000 men/year in 1998 [6]. In Arab populations, the incidence of prostate cancer correlates with low prostate volume and testosterone. Despite the low levels of testosterone, the aggressive forms of prostate cancer are found frequently in Arab patients, which indicate an increased sensitivity of Arab men to this steroid [7].

Prostate cancer is generally considered to be a complex disease with several genes underlying onset and severity, and minor susceptibility genes may play a larger role in prostate cancer risk. Recently, genome-wide association studies (GWAS) have identified dozens of common variants that confer susceptibility to prostate cancer across various ethnicities from European, Asian and African ancestry [8,9]. However, little is known about the prostate cancer genetic susceptibility in populations of Arab ancestry. The genetic susceptibility to prostate cancer is variable among different populations [10]. The difference may highlight the impact of genetic background on disease risk between populations. For instance, several SNPs located in 8q24 have been implied to a different population susceptibility to prostate cancer [11,12]. These variants, located in c-Myc gene, reduced the risk of prostate cancer in Caucasian but not in African Americans, thus protective mechanisms that might reduce c-Myc expression in Caucasians do not exist in African Americans. Identification of the genetic variants in different populations may help us to better understand the genetic and molecular mechanism of prostate cancer.

With the aim to identify genetic variants associated with prostate cancer in Arab populations, we first conducted a GWAS with DNA samples from a Tunisian cohort. We further extended the study to evaluate potential associations of 11 SNPs, identified by GWAS, in a cohort from Arab ancestry living in Qatar and Saudi Arabia. Functional significance of the identified prostate cancer associated SNPs was assessed by *in-silico* analysis and mRNA quantification of certain adjacent genes.

## Materials and methods

### Ethics statement

The Institutional Review Boards of Weill Cornell Medical College in Qatar, the Hamad Medical Corporation, and King Khalid University Hospital approved the study protocols. All subjects signed informed consent documents for participation in the study.

### Subjects

A total of 221 unrelated Tunisian men consisting of 90 PCa patients and 131 male age-matched controls were selected from the same population living in the middle coast of Tunisia. PCa patients were recruited from the two departments of Urology of Monastir and Sousse Hospital, Tunisia, in whom the diagnosis has been confirmed histologically. The serum prostate-specific antigen (PSA) values were measured in all the cases before treatment. Clinical characteristics including Gleason grade, TNM stage, age at diagnosis, and family history were obtained from medical records. The pathological stage at the time of diagnosis was classified according to TNM system into the localized group (T1− T2N0M0) and the advanced group (T3 − T4N0 M0 and T1 − T4N0–1 M1/T1− T4N1M0–1). Histopathological grade was recorded as the Gleason score and was classified into two groups: the low-grade group (Gleason score < 7) and the high-grade group (Gleason score7). A detailed description of the clinical-pathological characteristics of this cohort is summarized in Table 1.

**Table 1:** Description of the subjects for GWAS study

**Case number**	**90**
Age (mean ± SD)	73.7 ± 8.4^a^
PSA (mean ± SD)	225.5 ± 368.9
**Characteristic**	**Number (%)**
Tumor Stage	
T1-T2	17 (18.9)
T3-T4	44 (48.9)
Data missing	29 (32.2)
Lymph node	
Negative	52 (57.8)
Positive	7 (7.8)
Data missing	31 (34.4)
Metastasis	
Negative	42 (46.7)
Positive	18 (20.0)
Data missing	25 (33.3)
Gleason Score	
GS ≥ 7	44 (48.9)
GS < 7	19 (21.1)
Data missing	27 (30.0)

^a^ Age (mean ± SD) for controls: 59.7 ± 12.6.

The control group consisted of 131 healthy male subjects having no evidence of any personal or family history of cancer. Their PSA levels were within the normal limit (<4 ng/ml) and showed no signs of prostate hyperplasia or PCa by digital rectal examination. Those who had other known malignancies were also excluded.

There were 155 unrelated PCa patients and 182 age-matched controls from Arab ancestry living in Qatar and Saudi Arabia included in the replication genotyping study.

### DNA and RNA extraction

Genomic DNA was extracted from peripheral blood samples using QIAamp^®^ DNA blood Maxi Kit according to the manufacturer’s protocol (Qiagen, Valencia, CA). Thirty-six RNA samples were extracted from archived formalin-fixed paraffin-embedded non-malignant prostate tissues using RecoverAll™ Total Nucleic Acid Isolation Kit (Ambion, Grand Island, NY).

### Genotyping

Genome-wide scanning was applied with Affymetrix Genome-Wide Human SNP Array 6.0 following the manufacturer’s protocol. After genotype calling using the Birdsuite [13], the total SNPs on Affymetrix arrays were subjected to quality control. Nineteen samples were excluded due to low call rate (<95%). SNPs were excluded with low minor allele frequency (<5%), low call frequency (<95%) and replication error. The final SNP set included 534,781 SNPs for genome-wide association analysis.

The replication study was performed with the TaqMan^®^ SNP genotyping assays on a 7500 Real-Time PCR System (Applied Biosystems, Grand Island, NY), with no template as negative controls. The PCR thermal cycling was as follows: initial denaturing at 95°C for 10 min, 40 cycles of 92°C for 15 s and 60°C for 1 min. Genotype call success rate for cases and for controls was 94.8% and 98.2% respectively. Randomly selected 47 samples were verified genotype reproducibility with a coincidence rate 100%.

### Genotyping data analysis

To stratify and correct the population, we performed a multidimensional scaling (MDS) analysis as implemented in PLINK 1.07 [14] on the identity-by-state (IBS) matrix of our samples. After removing 7 outliers by plotting the main axes of variation against each other (Additional file 1 Figure S1), we use PLINK to analyze the genome-wide association with 498,148 autosomal SNPs and to perform permutation test to examine the stability of *P* values. Genotype distributions between cases and controls were evaluated by the chi-square test. Associations between genotypes/alleles and prostate cancer risk were estimated by computing odds ratios (ORs) and the corresponding 95% confidence intervals (CI) from unconditional logistic regression. Homozygotes for non-risk allele were the reference group, and then heterozygotes and homozygous risk allele genotypes were compared with the reference group, respectively. The linkage disequilibrium analyses were applied with Haploview [15].

### eQTL analysis

All the identified consecutive loci on chromosome 9, 17 and 22, were tested for correlation with nearby gene expression using the eQTL database Genevar [16]. Genotype and expression data within this database are derived from 3 cell types (fibroblast, lymphoblastoid cell line and T cell) from 75 individuals from Geneva, Switzerland [17] and 3 tissue types (166 adipose, 156 lymphoblastoid cell line and 160 skin) from healthy female twins [18]. The expression probes located within 1 Mb of the 5’ and 3’ end of the specified SNPs were analyzed. Differences in the distribution of normalized expression levels between genotypes were compared using a linear regression model. To avoid false positive associations due to multiple tests, we set a significance threshold of *P* < 1.0 × 10^−3^ and also assessed significance using 10,000-fold permutations (Perm).

### Gene expression quantification

Using GoTaq^®^ 2-Step RT-qPCR System, total RNA was prepared from 36 non-malignant prostate tissues and reverse transcribed and the mRNA of genes adjacent to the GWAS prostate cancer SNPs were quantified. Expression values were calculated as 2^-ΔCt^ using GAPDH gene as reference. Primer sequences are listed in the Additional file 2 Table S1. The difference in gene expression between prostate tumors carrying GWAS prostate cancer risk alleles and that without was calculated using T test with Welch’s correction.

## Results

**Figure 1:**
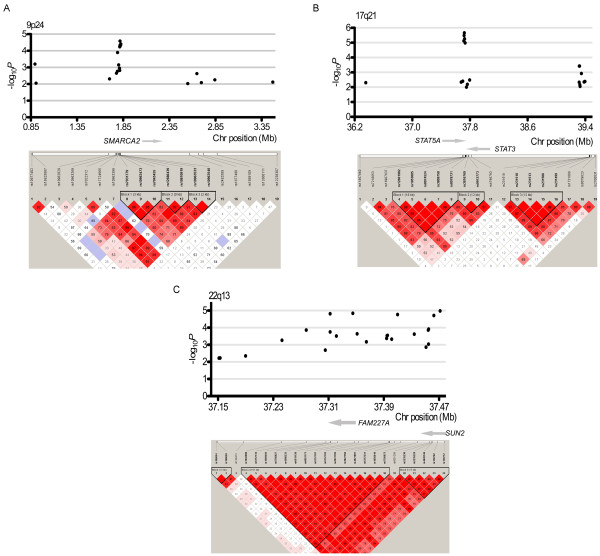
LD map of the SNPs and association results for the loci on the consecutive region of chromosome (A)9, (B)17 and (C)22.

Genome Scan analysis of DNA from 90 Tunisian patients with prostate cancer and 131 age-matched control subjects revealed association with 5,749 SNPs (*P* < 0.01) (Additional file 1 Figure S2 and Additional file 3 Table S2). The genotype distributions of all these SNPs were in Hardy-Weinberg equilibrium (*P* > 0.05, the related data of 14 SNPs on chromosome 9, 17 and 22 are presented in Additional file 4 Table S3). Three consecutive regions were identified on chromosome 9, 17 and 22 encompassing 14 SNPs, which were found highly associated with prostate cancer (*P* = 1 × 10^-4^ to *P* = 1 × 10^-5^) (Table 2). Considering our modest sample size, we performed a permutation test of our GWAS dataset and these 14 SNPs still showed significant association (Additional file 5 Table S4). The LD structures of the SNPs (with *P* < 0.01) located in these 3 consecutive regions are shown in Figure 1.

**Table 2:** Fourteen SNPs identified by GWAS associated with prostate cancer risk in Tunisians

**dbSNP ID**	**Chr**	**Gene**	**Allele**^**a**^	**Risk allele frequency**		**HetOR(95%CI)**	***P***^***b***^	**HomOR(95%CI)**	***P***	**Risk allele OR(95%CI)**	***P***
				**Case**	**Control**						
Previous reported											
rs1053005	17	STAT3	T/C	0.45	0.26	2.94(1.30-4.41)	4 × 10^-3^	4.22(1.84-9.69)	4 × 10^-4^	**2.30(1.54-3.45)**	4 × 10^-5^
rs8074524	17	STAT3	C/T	0.45	0.25	2.43(1.32-4.47)	4 × 10^-3^	**5.05(2.14-12.0)**	1 × 10^-4^	**2.46(1.64-3.70)**	1 × 10^-5^
rs3809758	17	STAT3	C/T	0.45	0.26	2.25(1.23-4.14)	8 × 10^-3^	**4.41(1.90-10.2)**	3 × 10^-4^	**2.31(1.54-3.47)**	4 × 10^-5^
Newly reported											
rs7045455	9	SMARCA2	T/C	0.94	0.80	3.80(0.20-71.9)	0.2	12.5(0.70-222.6)	0.02	**3.98(1.97-8.05)**	4 × 10^-5^
rs12686439	9	SMARCA2	G/A	0.92	0.77	4.39(0.64-81.6)	0.16	13.2(0.74-234.5)	0.02	**3.32(1.80-6.15)**	7 × 10^-5^
rs10810919	9	SMARCA2	T/C	0.95	0.82	2.09(0.10-41.8)	0.35	9.14(0.50-167.8)	0.04	**5.03(2.22-11.4)**	2 × 10^-5^
rs10963533	9	SMARCA2	T/C	0.95	0.81	2.46(0.13-48.3)	0.31	9.30(0.51-170.9)	0.04	**4.19(2.01-8.75)**	5 × 10^-5^
rs10963540	9	SMARCA2	G/A	0.95	0.81	2.66(0.14-51.9)	0.28	11.0(0.61-198.6)	0.03	**4.81(2.23-10.4)**	2 × 10^-5^
rs12601982	17	STAT5A	A/G	0.35	0.17	2.27(1.25-4.14)	7 × 10^-3^	**6.43(2.15-19.3)**	3 × 10^-3^	**2.61(1.67-4.07)**	2 × 10^-5^
rs8078731	17	STAT3	A/T	0.32	0.16	2.06(1.10-3.84)	0.02	**5.67(1.85-17.4)**	1 × 10^-3^	**2.47(1.55-3.93)**	1 × 10^-4^
rs5750627	22	LOC646851	C/T	0.71	0.50	1.76(0.77-4.01)	0.17	**4.50(1.98-10.3)**	2 × 10^-4^	**2.40(1.60-3.60)**	2 × 10^-5^
rs6001173	22	LOC646851	C/T	0.70	0.50	1.84(0.81-4.18)	0.14	**4.30(1.89-9.78)**	3 × 10^-4^	**2.32(1.54-3.49)**	4 × 10^-5^
rs138702	22	SUN2	T/A	0.75	0.55	**3.14(1.12-8.86)**	0.02	**6.59(2.33-18.4)**	1 × 10^-4^	**2.44(1.60-3.71)**	3 × 10^-5^
rs138712	22	SUN2	A/G	0.74	0.54	2.54(0.96-6.72)	0.05	**5.92(2.25-15.6)**	1 × 10^-4^	**2.47(1.63-3.76)**	2 × 10^-5^

Note: Chr, chromosome; OR, Odds ratio; CI, confidence interval; HetOR, odds ratio in heterozygote; HomOR, odds ratio in homozygote for risk allele (relative to homozygote for non-risk allele).

^a^ Reference allele/risk allele.

^b^ Bold font indicates that *P* value reaches the adjusted significant level (i.e., 0.05/14).

To investigate whether the identified SNPs associated with prostate cancer in other Arab populations, we replicated the genotyping of 11 SNP in 337 subjects from Arab ancestry living in Qatar and Saudi Arabia (155 PCa patients and 182 age-matched control subjects). None of these SNPs was found to be associated with prostate cancer risk in this distinct Arab population (Additional file 6 Table S5).

To address the functional significance of the 3 clusters of SNPs, we investigated the potential correlation between these SNPs and the expression of the adjacent genes. This includes: *SMARCA2* (SWI/SNF related, matrix associated, actin dependent regulator of chromatin, subfamily a, member 2) on chromosome 9, *STAT5A* (signal transducer and activator of transcription 5A) and *STAT3* (signal transducer and activator of transcription 3) on chromosome 17 and *FAM227A* (family with sequence similarity 227, member A) and *SUN2* (Sad1 and UNC84 domain containing 2) on chromosome 22. *In-silico* analysis using eQTL database Genevar showed no correlation between prostate cancer-identified SNPs and the expression of the above described adjacent genes. We next expanded our search to the 2 Mb region flanking these SNPs. The cluster of SNPs on chromosome 17 are associated with the expression of *STAT5B* gene, which encodes a signal transducer and activator of transcription 5B, and the risk alleles were consistently associated with increased expression of *STAT5B* (Additional file 1 Figure S3A). On chromosome 22, rs5750627 and rs6001173 SNPs are associated with the expression of *APOBEC3H* (apolipoprotein B mRNA editing enzyme, catalytic polypeptide-like 3H), *CBX6* (chromobox homolog 6), *DDX17* (DEAD box helicase 17), *PLA2G6* (phospholipase A2, group VI), *KDELR3* (Lys-Asp-Glu-Leu endoplasmic reticulum protein retention receptor 3) and *CBY1* (chibby homolog 1, Drosophila), whereas rs138702 SNP is found to be associated with *APOBEC3H* (Additional file 1 Figure S3B), *JOSD1* (Josephin domain containing 1), *KDELR3* and *CBY1*. rs138712 is associated with the expression of *APOBEC3H*, *CBX6, PLA2G6* and *SUN2* (Additional file 1 Figure S3C). No correlation was found between GWAS prostate cancer SNPs located on chromosome 9 and the expression of nearby genes in the 2 Mb range.

**Figure 2:**
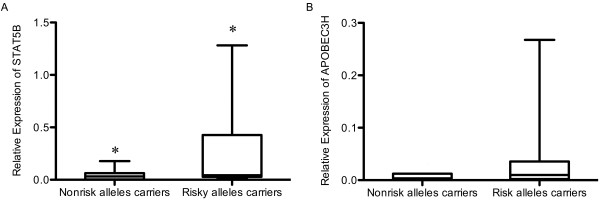
**The correlation between risk alleles and nearby gene expression.** (**A**) STAT5B mRNA expression grouped by risk alleles carriers and nonrisk alleles carriers. The individuals who carry at least 3 risk alleles out of the 5 SNPs on chromosome 17 are enrolled in risk alleles carriers group. The rest are enrolled in nonrisk alleles carriers group. (**B**) APOBEC3H mRNA expression grouped by risk alleles carriers and nonrisk alleles carriers. The individuals who carry 4 risk alleles of all those 4 SNPs on chromosome 22 are enrolled in risk alleles carriers grope. The rest are enrolled in nonrisk alleles carriers group includes the rest. (*P = 0.011 and *P* = 0.080 for Figure 2B).

To confirm the results obtained by *in-silico* analysis, we assessed the mRNA levels of the above-described genes in 36 non-malignant prostate tissues of patients. Samples carrying more than three risk alleles of chromosome 17 SNPs (rs12601982, rs1053005, rs8074524, rs3809758 and rs8078731) showed significantly higher expression of *STAT5B* (Figure 2A, 0.282 ± 0.083 N = 22 vs. 0.049 ± 0.016 N = 14, *P* = 0.011). We also examined the mRNA levels of 4 genes located on chromosome 22: *APOBEC3H*, *CBY1, DDX17* and *JOSD1*. We observed higher levels of *APOBEC3H* mRNA in prostate samples carrying risk alleles of the GWAS prostate cancer SNPs (Figure 2B) but the difference did not reach statistical significance (0.043 ± 0.020 N = 18 vs. 0.006 ± 0.001 N = 14, *P* = 0.080. No APOBEC3H amplification in the other 4 samples). For the remaining chromosome 22 genes, no correlation was found between GWAS prostate cancer SNPs and their mRNA levels.

## Discussion

With the fulfillment of the GWAS in the search of alleles that predispose Tunisians for the prostate cancer, we identified 3 genomic regions containing 14 SNPs, 11 of which were newly reported risky loci associated with prostate cancer (Table 2). The region 17q21 containing *STAT3* was reported associated with prostate cancer in the Caucasian population [19]. rs1053005 and rs3809758 (r^2^ = 0.93), located in 17q21, were independently associated with more aggressive PCa [20]. Similarly, rs8074524 in perfect LD with rs3809758 (r^2^ = 1.00 in the Caucasian HapMap population) was associated with risk of more aggressive prostate cancer [20]. In the Tunisian population, LD among these 3 SNPs is close to perfect (all r^2^ > 0.95, Figure 1). rs12601982 residing in the intron of the *STAT5A* gene is located in the same LD block as the reported loci (r^2^ = 0.98), suggesting that this region may be a promising common prostate cancer marker in different ethnic populations.

The most direct way to address the genetic effects of SNPs is to evaluate their effects on the transcription of nearby genes or genes within 1 Mb 5’ or 3’ direction. From our dataset, we did not find any correlation between the SNPs on chromosome 17 and *STAT3* or *STAT5A* expression levels. However, both the publicly available data analysis and experimental results indicated a correlation between the SNPs on chromosome 17 and *STAT5B* expression. The clinical and functional implications of transcription factor *STAT5A/B* and *STAT3* have been well established in prostate cancer. It has been shown that they are constitutively active in both locally confined and advanced prostate cancer and critical for the growth of PC cells [21-26]. Furthermore, *STAT5A/B* and *STAT3* have potential importance in the promotion of metastasis of PC cells [27-29]. Interestingly, it seems that *STAT5B* particularly played a major role in cancer cell viability and growth [29]. In spite of our small cohort, we showed that the chromosome 17 prostate cancer risk alleles are connected with higher expression of *STAT5B*. This locus may represent a very promising molecular marker for prostate cancer diagnostics and prognostics in different ethnic populations.

The 5 SNPs on 9p24 are mapped to upstream of the *SMARCA2* gene. The encoded protein of this gene is part of the large ATP-dependent chromatin remodeling complex SNF/SWI, which is required for transcriptional activation of genes normally repressed by chromatin. SMARCA2 and/or its binding protein SMARCA4 is absent or disrupted in approximately 17% of all human adenocarcinomas [30]. Down-regulation of SMARCA2 expression was found in prostate cancer tissues and conferred the proliferation advantage to prostate cancer cells [31]. 9p24 was linked to ovarian cancer and colorectal cancer [32,33]**.** To our knowledge this is the first time that 9p24 region has been associated to the risk of prostate cancer.

On chromosome 22, rs5750627 and rs6001173 are located in the intron region of the *FAM227A* gene that encodes a hypothetical protein LOC646851. A region of deletion on chromosome 22q13 is common to human breast and colorectal cancers [34], suggesting the existence of putative tumor suppressor gene(s) at this location. Two 22q13 regions with marker D22S445 and D22S274, respectively, are shown to be associated with aggressive form of prostate cancer [35,36], and these two regions are far from the region we have identified. rs138702 and rs138712 are located in the intron region of the *SUN2* gene. *SUN2* encodes an inner nuclear membrane protein that plays a major role in nuclear-cytoplasmic connection. In mouse, loss of SUN2 increases the sensitivity to DNA damage [37]. There is no evidence showing that SUN2 is related to onset and development of cancer. In the present study, we showed that prostate samples carrying homozygous risk alleles of rs5750627, rs6001173 and rs138702 have a trend of higher expression of *APOBEC3H*. This protein belongs to the apolipoprotein B mRNA-editing enzyme catalytic polypeptide 3 families and encodes a cytidine deaminase, which leads to G to A RNA editing. Although *APOBEC3* family is implicated in resistance to gamma-retrovirus XMRV to reduce prostate cancer [38], there is evidence that *APOBEC3* family inhibit miRNA activity to promote cancer metastasis [39], which represents a more general mechanism involved in cancer. A meta-analysis of four GWAS identified a novel PCa susceptibility locus, rs11704416, which also locates on 22q13 [40]. However, rs11704416 is not associated with PCa risk in our cohort. This meta-analysis illustrated the value of combining GWAS, which prompts us to collect more PCa GWAS data of Arabs to identify further susceptibility alleles in future study.

Arabs refer to people originally living in Western Asia and North Africa. They share similar genealogical, linguistic or cultural grounds. Recently, several studies reflected the genetic makeup of Arab population and revealed the diversity among the ethnic groups [41-43]. Although analyses based on classical genetic markers did not show discernable difference between different Berber- (North African origin) and Arabic- (Western Asia origin) speaking populations [41], mitochondrial and genome-wide SNP genotyping data clearly indicate a back-to-Africa (east-to-west) gene flow attribute [42]. SNPs usually occur in ethnic-specific patterns. Only a small number of the risky variants identified in other populations like Caucasian [11], African American [12], Japanese [44] and Han [45] can be replicated in the Tunisian population. According to the migration study of North Africans [42], Tunisians have the highest proportion of Maghrebi ancestry, with or without little evidence of admixture of West Asian population. The genetic structure differences between Tunisians and West Asians may explain the difficulty replicating the findings of the Tunisian GWAS prostate cancer study with the population of Arab ancestry living in Saudi Arabia and Qatar. Our observation highlighted the ethnic heterogeneity of genetic mechanisms of prostate cancer.

The present study based on a small-scale GWAS of prostate cancer in Tunisians led to the identification of 3 clusters of SNPs in strong linkage disequilibrium with high odd-ratios (OR > 2). When they are well designed small-scale GWAS can lead to significant findings. For instance, the search of genes underlying the age-related macular degeneration undertaken with a small-scale GWAS using DNA samples from only 96 cases and 50 control subjects led to a prominent finding showing that a variant in complement factor H gene has an important causative effect (OR = 7.4) [46]. A case–control study of American African men on the association of EphB2 SNPs with PCa risk suggests that the power of GWAS study relies on high resolution haplotype maps based on African population [47]. Further analysis of our GWAS study should be performed when a high-resolution genetic maps of Arabs is available.

Although this study, showing the strong association of SNPs located on chromosome 9, 17 and 22 with prostate cancer in Tunisians, suggests that genes in chromosomal regions play a role in susceptibility to the onset of prostate cancer, further studies are needed to clarify their role in the pathogenesis of the disease. Our data also suggest a need for large-scale prostate cancer associated SNPs typing in Tunisians with prostate cancer. Replication of our findings in other Arab North African populations will be of use in determining whether the relations between these risk factors and the development of prostate cancer can be generalized.

## Competing interests

The authors declare that they have no competing interests.

## Authors’ contributions

JS, KAR and LC designed the study and wrote the manuscript. KAR, DR, IAB, KF, SAS, HK and IIA were responsible for recruiting patients for the study. JS, DK and SPD generated the data. JS, KAR, DK, JR, HK, SJ and LC analyzed the data. All the authors read and approved the final manuscript.

## Supplementary Material

Additional file 1: Figure S1 Multidimensional scaling (MDS) analysis of sample variation pattern in 202 arrays of good quality. A total of 221 arrays have been hybridized, in which 202 passed the quality control criteria. (**A**) Two outliers were identified by PC1, three outliers by PC2, and (B) two outliers by PC4. **Figure S2**. Manhattan plot of the strength of association (−log10 (*P*) values; Y-axis) between SNPs (X-axis by chromosome and chromosomal position) and prostate cancer risk. SNPs on each individual chromosome are shown with the same color in an order from chromosome 1 to 22 (left → right). **Figure S3**. eQTL analysis. (**A**) The correlation between SNPs on chromosome 17 and mRNA expression of STAT5B in lymphoblastoid cells from 75 Geneva individuals. The mRNA was quantified by array probe ILMN_1777783. (**B**) The correlation between SNPs on chromosome 22 and mRNA expression of STAT5B in lymphoblastoid cells from 75 Geneva individuals. The mRNA was quantified by array probe ILMN_1664828. The data of rs6001173 and rs138172 are not available from the online data. (**C**) The correlation between SNPs on chromosome 22 and mRNA expression of SUN2 in 166 adipose (**A**), 156 lymphoblastold cell line (L) and 160 skin (S) samples, respectively, from healthy female twins (Twins 1 and 2). The mRNA was quantified by array probe ILMN_2099301. Click here for file

Additional file 2: Table S1 Primer sequences used in real-time quantitative PCR. Click here for file

Additional file 3: Table S2 The association of top 5749 SNPs identified by GWAS. Click here for file

Additional file 4: Table S3 GWAS PCa genotype distributions in patients and controls. Click here for file

Additional file 5: Table S4 The results of permutation test of fourteen PCa SNPs. Click here for file

Additional file 6: Table S5 The replication study in the population with Arab ancestry living in Qatar and Saudi Arabia (155 cases and 182 controls). Click here for file
