# Supplementary material for: Genome scan study of prostate cancer in Arabs: identification of three genomic regions with multiple prostate cancer susceptibility loci in Tunisians
Source: J Transl Med. 2013 May 13;11:121. doi: 10.1186/1479-5876-11-121 (PMC3659060; doi:10.1186/1479-5876-11-121)
Supplement: Additional file 1: Figure S1 — Multidimensional scaling (MDS) analysis of sample variation pattern in 202 arrays of good quality. A total of 221 arrays have been hybridized, in which 202 passed the quality control criteria. (A) Two outliers were identified by PC1, three outliers by PC2, and (B) two outliers by PC4. Figure S2. Manhattan plot of the strength of association (−log10 (P) values; Y-axis) between SNPs (X-axis by chromosome and chromosomal position) and prostate cancer risk. SNPs on each individual chromosome are shown with the same color in an order from chromosome 1 to 22 (left → right). Figure S3. eQTL analysis. (A) The correlation between SNPs on chromosome 17 and mRNA expression of STAT5B in lymphoblastoid cells from 75 Geneva individuals. The mRNA was quantified by array probe ILMN_1777783. (B) The correlation between SNPs on chromosome 22 and mRNA expression of STAT5B in lymphoblastoid cells from 75 Geneva individuals. The mRNA was quantified by array probe ILMN_1664828. The data of rs6001173 and rs138172 are not available from the online data. (C) The correlation between SNPs on chromosome 22 and mRNA expression of SUN2 in 166 adipose (A), 156 lymphoblastold cell line (L) and 160 skin (S) samples, respectively, from healthy female twins (Twins 1 and 2). The mRNA was quantified by array probe ILMN_2099301. [file 1479-5876-11-121-S1.docx]

Supplementary Figures

A


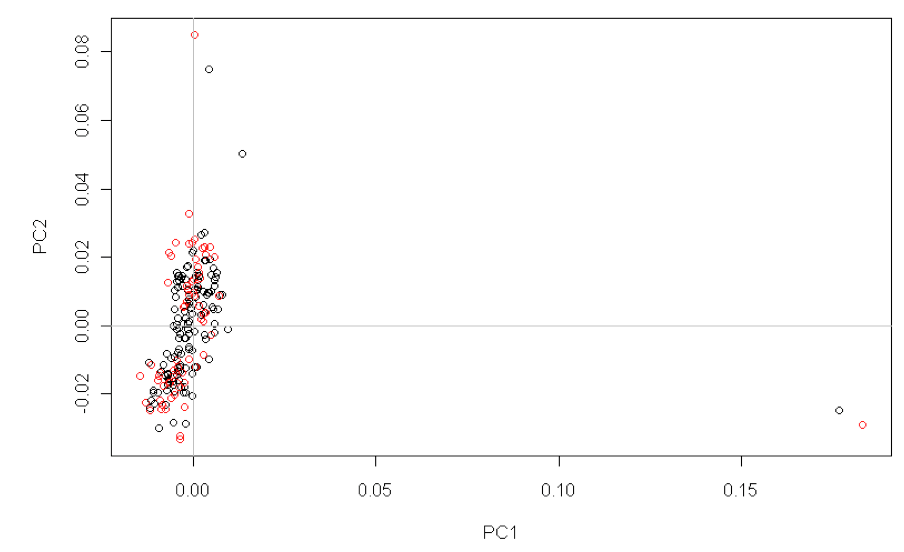


B


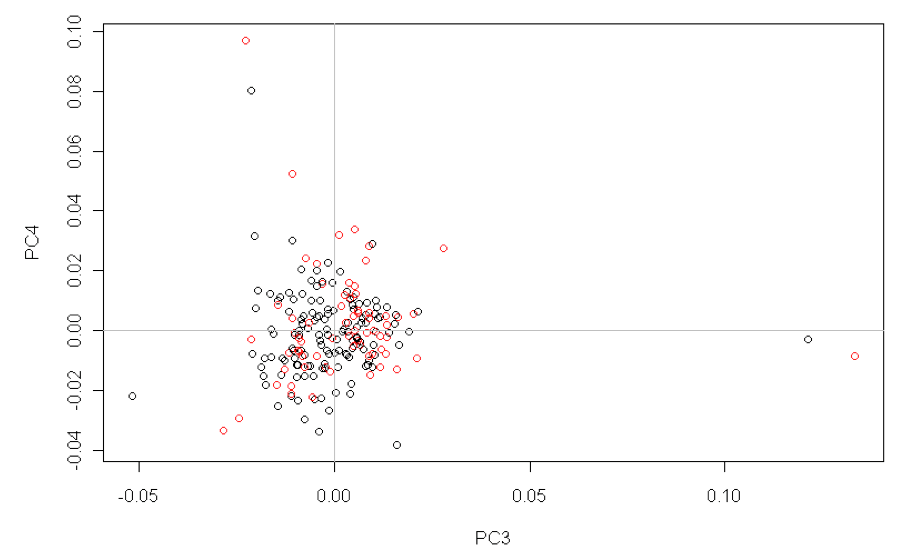


Figure S1. Multidimensional scaling (MDS) analysis of sample variation pattern in 202 arrays of good quality. A total of 221 arrays have been hybridized, in which 202 passed the quality control criteria. (A) Two outliers were identified by PC1, three outliers by PC2, and (B) two outliers by PC4.


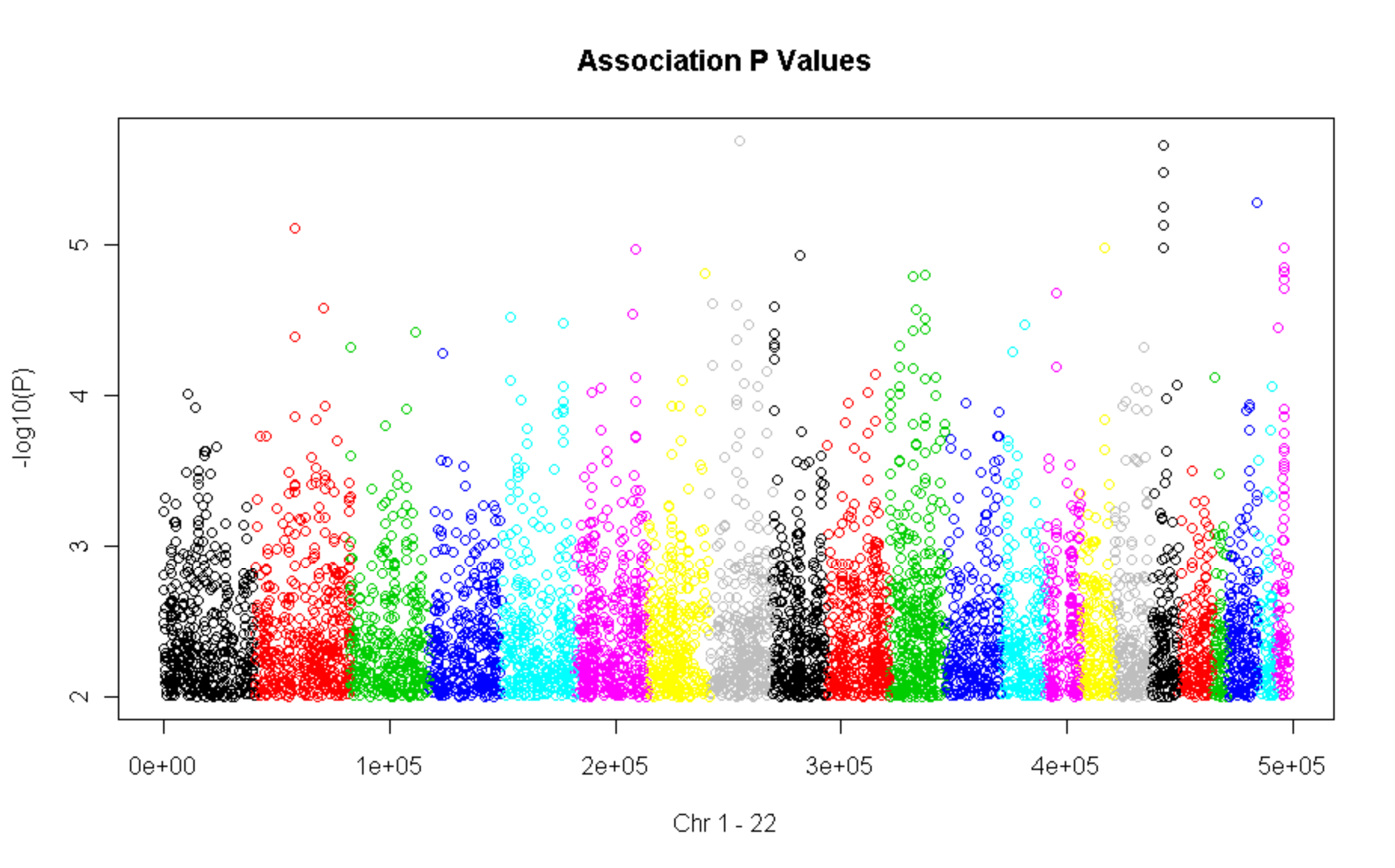


Figure S2. Manhattan plot of the strength of association (–log10 (*P*) values; Y-axis) between SNPs (X-axis by chromosome and chromosomal position) and prostate cancer risk. SNPs on each individual chromosome are shown with the same color in an order from chromosome 1 to 22 (left🡺right).


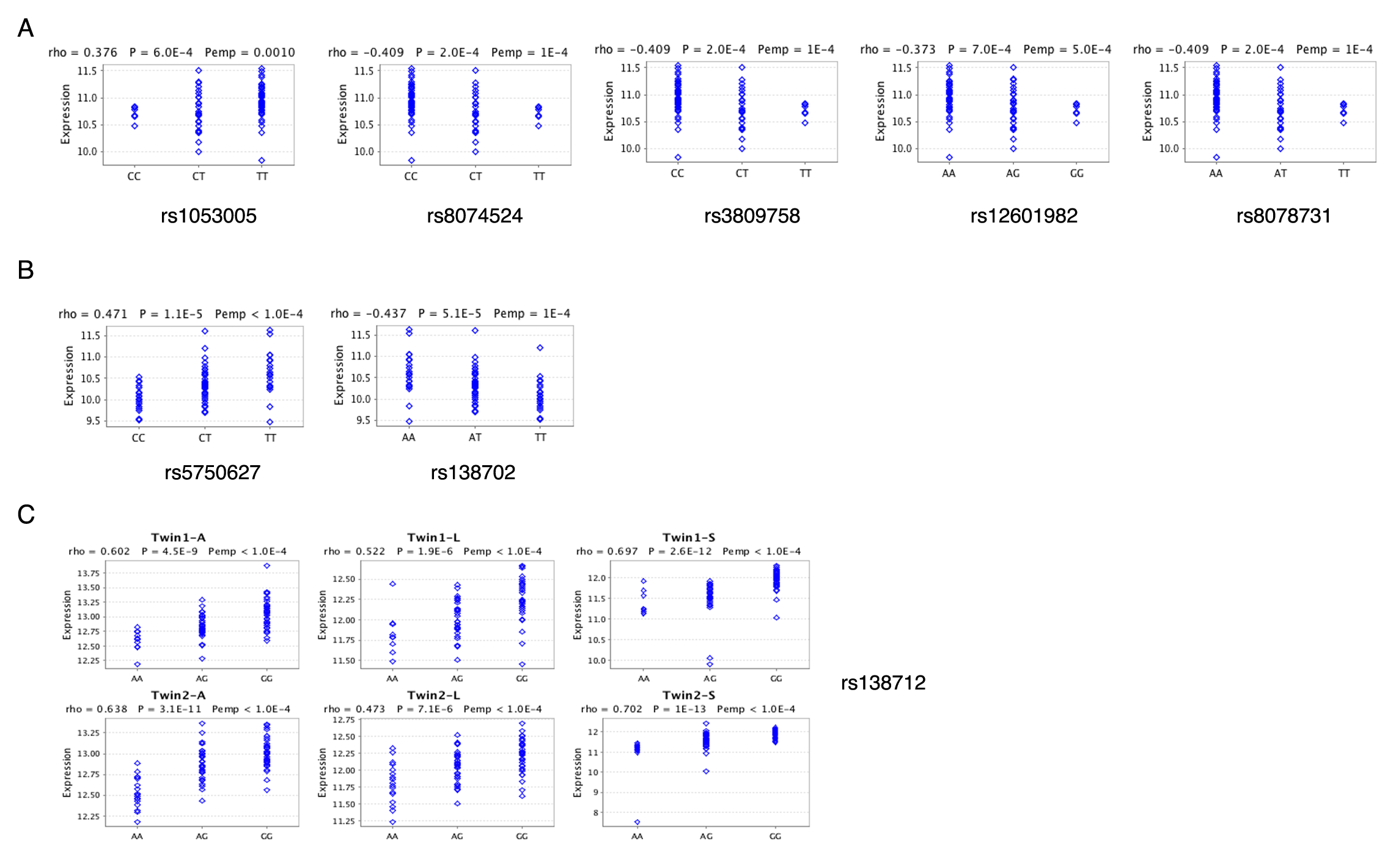


Figure S3. eQTL analysis. (A) The correlation between SNPs on chromosome 17 and mRNA expression of STAT5B in lymphoblastoid cells from 75 Geneva individuals. The mRNA was quantified by array probe ILMN_1777783. (B) The correlation between SNPs on chromosome 22 and mRNA expression of STAT5B in lymphoblastoid cells from 75 Geneva individuals. The mRNA was quantified by array probe ILMN_1664828. The data of rs6001173 and rs138172 are not available from the online data. (C) The correlation between SNPs on chromosome 22 and mRNA expression of SUN2 in 166 adipose (A), 156 lymphoblastold cell line (L) and 160 skin (S) samples, respectively, from healthy female twins (Twins 1 and 2). The mRNA was quantified by array probe ILMN_2099301.
