# Supplementary material for: Genome scan study of prostate cancer in Arabs: identification of three genomic regions with multiple prostate cancer susceptibility loci in Tunisians
Source: J Transl Med. 2013 May 13;11:121. doi: 10.1186/1479-5876-11-121 (PMC3659060; doi:10.1186/1479-5876-11-121)
Supplement: Additional file 2: Table S1 — Primer sequences used in real-time quantitative PCR. [file 1479-5876-11-121-S2.docx]

Table S1. Primer sequences used in real-time quantitative PCR.

| Primer | Sequence (5’-3’) |
| --- | --- |
| SMARCA2 forward | GAAGAGTCAGAGTCCGAGGCA |
| SMARCA2 reverse | TCGCTCACTACAGGTTTGGCT |
| STAT5A forward | GGCTCCCTATAACATGTACCC |
| STAT5A reverse | AAGACTGTCCATTGGTCGGCG |
| STAT5B forward | GTGAAGCCACAGATCAAGCA |
| STAT5B reverse | TACGTCCATTGTGTCCTCCA |
| STAT3 forward | GAGGACTGAGCATCGAGCA |
| STAT3 reverse | CATGTGATCTGACACCCTGAA |
| FAM227A forward | tcaatgaggaatcacctgac |
| FAM227A reverse | tcagggcttggaagtgagtg |
| SUN2 forward | agacgtttcactttcaggcc |
| SUN2 reverse | agcggtagatgcaggtgtac |
| APOBEC3H forward | tgtggaccacgagaaaccg |
| APOBEC3H reverse | atccatgtaacgaccctgcg |
| DDX17 forward | ccgctcccagatctctat |
| DDX17 reverse | caccacctcttgctccaa |
| JOSD1 forward | acgccctcaataacgtctTC |
| JOSD1 reverse | aggtgtcaccatggtgtttg |
| CBY1 forward | catctctctccaacctgca |
| CBY1 reverse | acttcaggctttgccctg |
| GAPDH forward | GAAGGTGAAGGTCGGAGTCAAC |
| GAPDH reverse | CAGAGTTAAAAGCAGCCCTGGT |
