# Supplementary material for: Genome scan study of prostate cancer in Arabs: identification of three genomic regions with multiple prostate cancer susceptibility loci in Tunisians
Source: J Transl Med. 2013 May 13;11:121. doi: 10.1186/1479-5876-11-121 (PMC3659060; doi:10.1186/1479-5876-11-121)
Supplement: Additional file 4: Table S3 — GWAS PCa genotype distributions in patients and controls. [file 1479-5876-11-121-S4.docx]

Table S3. GWAS PCa genotype distributions in patients and controls

| **SNP** | **Chr** | **Genotype** | **Patients(n)** | ***P* for HWE** | **Controls(n)** | ***P* for HWE** |
| --- | --- | --- | --- | --- | --- | --- |
| rs7045455 | 9 | T/T | 0 | 1 | 7 | 0.42 |
|  |  | T/C | 10 |  | 41 |  |
|  |  | C/C | 75 |  | 90 |  |
| rs12686439 | 9 | G/G | 0 | 1 | 7 | 1 |
|  |  | G/A | 14 |  | 49 |  |
|  |  | A/A | 71 |  | 81 |  |
| rs10810919 | 9 | T/T | 0 | 1 | 5 | 0.77 |
|  |  | T/C | 7 |  | 39 |  |
|  |  | C/C | 78 |  | 94 |  |
| rs10963533 | 9 | T/T | 0 | 1 | 5 | 1 |
|  |  | T/C | 9 |  | 42 |  |
|  |  | C/C | 76 |  | 90 |  |
| rs10963540 | 9 | G/G | 0 | 1 | 6 | 0.59 |
|  |  | G/A | 8 |  | 41 |  |
|  |  | A/A | 77 |  | 91 |  |
| rs1053005 | 17 | T/T | 27 | 0.51 | 13 | 0.19 |
|  |  | T/C | 39 |  | 47 |  |
|  |  | C/C | 19 |  | 78 |  |
| rs8074524 | 17 | C/C | 27 | 0.51 | 11 | 0.36 |
|  |  | C/T | 39 |  | 47 |  |
|  |  | T/T | 19 |  | 79 |  |
| rs3809758 | 17 | C/C | 28 | 0.38 | 78 | 0.26 |
|  |  | C/T | 38 |  | 47 |  |
|  |  | T/T | 19 |  | 12 |  |
| rs8078731 | 17 | A/A | 38 | 0.13 | 98 | 0.36 |
|  |  | A/T | 28 |  | 35 |  |
|  |  | T/T | 11 |  | 5 |  |
| rs12601982 | 17 | A/A | 38 | 0.24 | 94 | 0.55 |
|  |  | A/G | 34 |  | 37 |  |
|  |  | G/G | 13 |  | 5 |  |
| rs5750627 | 22 | C/C | 10 | 0.19 | 37 | 0.39 |
|  |  | C/T | 30 |  | 63 |  |
|  |  | T/T | 45 |  | 37 |  |
| rs6001173 | 22 | C/C | 10 | 0.24 | 38 | 0.2 |
|  |  | C/T | 30 |  | 62 |  |
|  |  | T/T | 43 |  | 38 |  |
| rs138702 | 22 | T/T | 5 | 1 | 30 | 0.49 |
|  |  | T/A | 33 |  | 63 |  |
|  |  | A/A | 47 |  | 43 |  |
| rs138712 | 22 | A/A | 6 | 0.78 | 41 | 0.61 |
|  |  | A/G | 32 |  | 65 |  |
|  |  | G/G | 47 |  | 31 |  |
