# Supplementary material for: Genome scan study of prostate cancer in Arabs: identification of three genomic regions with multiple prostate cancer susceptibility loci in Tunisians
Source: J Transl Med. 2013 May 13;11:121. doi: 10.1186/1479-5876-11-121 (PMC3659060; doi:10.1186/1479-5876-11-121)
Supplement: Additional file 5: Table S4 — The results of permutation test of fourteen PCa SNPs. [file 1479-5876-11-121-S5.docx]

Table S4. The results of permutation test of fourteen PCa SNPs

| CHR | SNP | EMP1^a^ | NP^b^ |
| --- | --- | --- | --- |
| 9 | rs7045455 | 0.000402 | 104475 |
| 9 | rs12686439 | 0.000089 | 472000 |
| 9 | rs10810919 | 0.000247 | 169830 |
| 9 | rs10963533 | 0.000271 | 155000 |
| 9 | rs10963540 | 0.000327 | 128355 |
| 17 | rs12601982 | 0.000007 | 1000000 |
| 17 | rs1053005 | 0.000019 | 1000000 |
| 17 | rs8074524 | 0.000002 | 1000000 |
| 17 | rs3809758 | 0.000021 | 1000000 |
| 17 | rs8078731 | 0.000007 | 1000000 |
| 22 | rs5750627 | 0.00006 | 700299 |
| 22 | rs6001173 | 0.000098 | 430000 |
| 22 | rs138702 | 0.000065 | 644355 |
| 22 | rs138712 | 0.000037 | 1000000 |

^a^ Empirical *P* value

^b^ Number of permutations
