# Supplementary material for: Genome scan study of prostate cancer in Arabs: identification of three genomic regions with multiple prostate cancer susceptibility loci in Tunisians
Source: J Transl Med. 2013 May 13;11:121. doi: 10.1186/1479-5876-11-121 (PMC3659060; doi:10.1186/1479-5876-11-121)
Supplement: Additional file 6: Table S5 — The replication study in the population with Arab ancestry living in Qatar and Saudi Arabia (155 cases and 182 controls). [file 1479-5876-11-121-S6.docx]

Table S5. The replication study in the population with Arab ancestry living in Qatar and Saudi Arabia (155 cases and 182 controls)

|  |  |  |  | MAF | | Per Risky Allele OR |  |
| --- | --- | --- | --- | --- | --- | --- | --- |
| dbSNP ID | **Chr** | **Gene** | **Allele^a^** | **Case** | **Control** | **(95%CI)** | ***P*** |
| rs7045455 | 9 | SMARCA2 | T/C | 0.11 | 0.12 | 1.05(0.64-1.73) | 0.84 |
| rs12686439 | 9 | SMARCA2 | A/G | 0.09 | 0.1 | 1.18(0.69-2.02) | 0.55 |
| rs10963533 | 9 | SMARCA2 | C/T | 0.1 | 0.1 | 1.01(0.60-1.71) | 0.96 |
| rs12601982 | 17 | STAT5A | G/A | 0.31 | 0.28 | 1.14(0.81-1.61) | 0.44 |
| rs1053005 | 17 | STAT3 | C/T | 0.34 | 0.36 | 1.06(0.77-1.46) | 0.71 |
| rs8074524 | 17 | STAT3 | T/C | 0.34 | 0.32 | 1.07(0.77-1.49) | 0.69 |
| rs3809758 | 17 | STAT3 | T/C | 0.33 | 0.32 | 1.02(0.73-1.43) | 0.9 |
| rs8078731 | 17 | STAT3 | T/A | 0.31 | 0.29 | 1.10(0.79-1.54) | 0.57 |
| rs5750627 | 22 | LOC646851 | C/T | 0.27 | 0.3 | 1.16(0.82-1.62) | 0.4 |
| rs6001173 | 22 | LOC646851 | C/T | 0.3 | 0.32 | 1.07(0.77-1.51) | 0.68 |
| rs138702 | 22 | SUN2 | A/T | 0.23 | 0.26 | 1.13(0.78-1.63) | 0.52 |

Note: MAF, minor allele frequency; OR, odds ratio.

^a^ minor allele/major allele
